# Immunophenotyping of Peripheral Blood Mononuclear Cells in Septic Shock Patients With High-Dimensional Flow Cytometry Analysis Reveals Two Subgroups With Differential Responses to Immunostimulant Drugs

**DOI:** 10.3389/fimmu.2021.634127

**Published:** 2021-03-22

**Authors:** Ya Wang, Brian Gloss, Benjamin Tang, Suat Dervish, Brigitte Santner-Nanan, Christina Whitehead, Kristy Masters, Kristen Skarratt, Sally Teoh, Stephen Schibeci, Nicole Fewings, Chrystelle Brignone, Frederic Triebel, David Booth, Anthony McLean, Marek Nalos

**Affiliations:** ^1^Department of Intensive Care Medicine, Nepean Hospital, Penrith, NSW, Australia; ^2^Centre for Immunology and Allergy Research, The Westmead Institute for Medical Research, Sydney, NSW, Australia; ^3^Westmead Research Hub, Westmead Institute for Medical Research, Sydney, NSW, Australia; ^4^Westmead Cytometry, The Westmead Institute for Medical Research, Sydney, NSW, Australia; ^5^Charles Perkins Centre Nepean, Sydney Medical School Nepean, The University of Sydney, Kingswood, NSW, Australia; ^6^Department of Medicine, Faculty of Medicine and Health, Nepean Clinical School, The University of Sydney, Kingswood, NSW, Australia; ^7^Westmead Clinical School, University of Sydney, Sydney, NSW, Australia; ^8^Immutep S.A.S., Parc Club Orsay, Orsay, France; ^9^1st Department of Medicine, Medical Faculty in Plzen, Charles University, Prague, Czechia

**Keywords:** sepsis, high-dimensional flow cytometry, Lipopolysaccharides, interferon-γ, LAG-3Ig, immunophenotype

## Abstract

Sepsis is associated with a dysregulated inflammatory response to infection. Despite the activation of inflammation, an immune suppression is often observed, predisposing patients to secondary infections. Therapies directed at restoration of immunity may be considered but should be guided by the immune status of the patients. In this paper, we described the use of a high-dimensional flow cytometry (HDCyto) panel to assess the immunophenotype of patients with sepsis. We then isolated peripheral blood mononuclear cells (PBMCs) from patients with septic shock and mimicked a secondary infection by stimulating PBMCs for 4 h *in vitro* with lipopolysaccharide (LPS) with or without prior exposure to either IFN-γ, or LAG-3Ig. We evaluated the response by means of flow cytometry and high-resolution clustering cum differential analysis and compared the results to PBMCs from healthy donors. We observed a heterogeneous immune response in septic patients and identified two major subgroups: one characterized by hypo-responsiveness (Hypo) and another one by hyper-responsiveness (Hyper). Hypo and Hyper groups showed significant differences in the production of cytokines/chemokine and surface human leukocyte antigen-DR (HLA-DR) expression in response to LPS stimulation, which were observed across all cell types. When pre-treated with either interferon gamma (IFN-γ) or lymphocyte-activation gene 3 (LAG)-3 recombinant fusion protein (LAG-3Ig) prior to LPS stimulation, cells from the Hypo group were shown to be more responsive to both immunostimulants than cells from the Hyper group. Our results demonstrate the importance of patient stratification based on their immune status prior to any immune therapies. Once sufficiently scaled, this approach may be useful for prescribing the right immune therapy for the right patient at the right time, the key to the success of any therapy.

## Introduction

Sepsis is a life-threatening condition caused by a dysregulated inflammatory response to infection, which leads to multiple organ dysfunction. Mortality rates of patients suffering severe sepsis are estimated at 26%, and those with septic shock at 40%, contributing to around 5 million deaths a year globally (1, 2). The immune response during sepsis has been described as a biphasic event with an initial cytokine-mediated hyper-inflammatory phase aimed to eradicate the invading pathogens, and a subsequent immunosuppressed phase, as a result of prolonged activation of anti-inflammatory responses counteracting the initial pro-inflammatory responses (3, 4) but there is no clear delineation between the opposing phases (5). Importantly, a majority of septic patients, surviving the hyper-inflammatory phase often die from secondary or opportunistic infections during the immunosuppressed phase (6, 7).

Immune suppression in sepsis is characterized by lymphocyte apoptosis, anergy, a relative increase in T regulatory cells and myeloid derived suppressor cells and deficiencies in MHC class II mediated antigen presentation (8–13). These factors contribute to impaired antigen recognition, reduced antimicrobial effector functions, and poor microbial killing. Immunotherapies aimed at boosting immune responses, by enhancing antigen presentation capacity and lymphocytic functions, have shown some promising results (14–17). Interferon gamma (IFN-γ) has been trialed in sepsis patients and its use was associated with restoration of immune functions with increased HLA-DR expression in monocytes, increased production of cytokines such as tumor necrosis factor alpha (TNF-α), interleukin-1β (IL-1β) and decreased production of interleukin-6 (IL-6) and interleukin-10 (IL-10) (18, 19). The MHC Class II agonist LAG-3Ig is a soluble LAG-3 protein that activates antigen presenting cell (APC) leading to CD8^+^ T cell activation (20–22). Clinical effectiveness of LAG-3Ig (eftilagimod alpha) has been investigated in several Phase I and Phase II trials in cancer patients (23, 24), but not yet in patients with sepsis due to the presence of significant heterogeneity of patient's immune responses in sepsis and a lack of practical methods to reduce such heterogeneity (25).

In this study, we aimed to develop a method to reduce heterogeneity of patient immune phenotypes prior to the administration of immune therapy. To this end, we evaluated the immune status of patients with septic shock by assessing the proportions of major immune cell subset within PBMCs as well as their ability to produce cytokines/chemokine in response to LPS. Furthermore, we assessed the responses to immunomodulatory agents such as IFN-γ and LAG-3Ig. Our findings suggest immune heterogeneity in septic patients could be identified and quantified by using HDCyto and such quantification may assist subsequent administration of immune therapy.

## Materials and Methods

### Reagents and Antibodies

Fluorochrome-conjugated antibodies (as listed in Supplementary Table 1), BD Cytofix/Cytoperm™ Fixation/Permeabilization Solution set, human BD Fc block™, BD GolgiPlug™ and BD Horizon Brilliant Stain Buffer Plus were purchased from BD Biosciences (San Jose, CA). UltraComp eBeads™ Compensation Beads were purchased from ThermoFisher Scientific (Waltham, MA). Recombinant human IFN-γ was purchased from PeproTech (Rocky Hill, NJ). LAG-3Ig was kindly provided by Immutep Pty Ltd. Bovine Serum Albumin solution (35% in D.PBS) and LPS were purchased from Sigma Aldrich (St. Louis, MO).

### Study Population

Thirteen patients who were admitted to the intensive care unit (ICU) of the Nepean Hospital (Kingswood, NSW, Australia) between December 2017 and February 2019, were recruited to this study. The inclusion and exclusion criteria are as follows:

Inclusion criteria: (1) age ≥18 years old; (2) presence of shock, likely of infectious origin defined as day 1 Sequential Organ Failure Assessment (SOFA) score cardiovascular > 2; (3) presence of at least 2 organ dysfunctions as defined by aggregate day 1 or day 2 SOFA score >4; (4) no treatment limitations articulated prior to study enrolment.

Exclusion criteria: current or recent use of known immune suppression medication such as steroids (>5 mg prednisone or equivalent) methotrexate, mycophenolate or recent use of immune checkpoint inhibitors.

Ten age-matched healthy volunteers (age ≥18 years old) were also recruited as controls.

### Sample Collection and Processing

Venous blood was collected into tubes containing EDTA. For patients with sepsis, blood was collected on days 1–3 post ICU admission. PBMCs were prepared from whole blood by density gradient centrifugation using Ficoll-Paque Plus (Bio-strategy Pty Ltd) and cryopreserved. Briefly, whole blood was diluted with an equal volume of Dulbecco's Phosphate-Buffered Saline (D.PBS) (without Ca^2+^ and Mg^2+^) and layered on top of an equal volume of Ficoll-Paque Plus in a 50 ml Falcon tube. This was followed by centrifugation using a swinging-bucket rotor at 400 × g for 30 min at room temperature (RT) with the brake off. After centrifugation, PBMCs at the interphase between Ficoll-Paque Plus and plasma, was transferred into a new 50 ml Falcon tube and washed twice with D.PBS. Each wash was followed by centrifugation at 300 × g for 10 min. For cryopreservation, PBMC cell pellet after the final centrifugation was resuspended in fetal bovine serum (FBS) and then 20% Dimethyl Sulfoxide (DMSO) diluted in FBS was added dropwise to the cell suspension to make the final concentration of DMSO to 10%. One milliliter (ml) of cell suspension was transferred to 2 ml cryovial in a Corning® CoolCell™ Cell Freezing Container (Sigma-Aldrich), which was left at −80°C freezer overnight before transferring to liquid nitrogen for long-term storage. On the day of experiment, cryopreserved PBMCs were thawed and treated with DNase I to prevent cell clumping (26). Briefly, frozen PBMCs were thawed rapidly in a 37°C water bath, followed by washes first with RPMI1640 (Lonza) with 2% FBS and 10 mM HEPES, pH 7.0–7.6 and then with D.PBS. PBMCs were then treated with 100 units/ml DNase I in the presence of 5 mM MgCl_2_ for 15 min at RT, during which cells were counted with the CytoFLEX Flow Cytometer (Beckman Coulter, Brea, CA). After DNase I treatment, cells were washed once with D.PBS and then resuspended in cell culture medium (RPMI1640 with 10% FBS, 2 mM L-Glutamine, 50 units/ml Penicillin/50 μg/ml Streptomycin at 1 × 10^6^ cells/ml) for culturing in BD Falcon® 5 ml polypropylene round bottom tube (*in vitro* Technologies) at 37°C, 5% CO_2_.

### *Ex vivo* Stimulation for Cytokine Detections

PBMCs were cultured under three conditions (two aliquots per condition and up to 1 × 10^6^ per aliquot): untreated, plus IFN-γ (100 ng/ml) (27) or plus LAG-3Ig (1 μg/ml) (22) for 2 h. BD GolgiPlug (1 μg/ml) for detection of intracellular cytokines was added to all the cultures which were then cultured with or without LPS (100 ng/ml) (27) for a further 4 h. Cells were harvested by pipetting at the end of stimulations and subjected to cell count using the CytoFLEX Flow Cytometer before proceeding with flow cytometry staining.

### Flow Cytometry Staining

After 6 h of culture, ~1 × 10^6^ PBMCs were washed twice by resuspending cells in 4 ml D.PBS and centrifugation at 300 × g, RT for 5 min. Washed cells were then transferred into a 96-well V-bottom plate for the following staining procedure. Cells were first stained with 100 μl of BD Horizon™ Fixable Viability Stain 440 UV diluted in D.PBS (1:1,000), for 10–15 min at RT in the dark as per manufacturer's instructions. Cells were washed twice with 250 μl of staining buffer (D.PBS with 0.5% Bovine Serum Albumin) with centrifugation as above before surface staining. For surface staining, cells were incubated with 2.5 μg of human Fc Block diluted in staining buffer for 10 min at RT. Fluorochrome-conjugated surface antibodies as listed in Supplementary Table 1 (amount of antibody used for each test was pre-titrated) was then added along with BD Horizon™ Brilliant Stain Buffer Plus (10 μl per test) in a final staining volume to 100 μl. Samples were incubated for 30 min at 4°C in the dark, then were washed four times with 250 μl staining buffer with centrifugation as above. Washed cell pellets were fixed and permeabilized with 100 μl BD Cytofix/Cytoperm™ Fixation/Permeabilization Solution for 20 min at 4°C in the dark. Fixation/Permeabilizaiton buffer was removed by two washes with 250 μl of freshly prepared 1 × BD Perm/Wash buffer. All centrifugation steps after permeabilization were performed at 500 × g for 5 min at RT. The cell pellet was resuspended in 200 μl of staining buffer and stored overnight at 4°C in the dark. The following day, cells were pelleted by centrifugation (500 × g, 5 min, RT) and fixed/permeabilized again as described above. For intracellular staining, cells were incubated with 100 μl of fluorochrome-conjugated antibody mixture as listed in Supplementary Table 1 (amount of antibody used for each test was pre-titrated) diluted in 1 × BD Perm/Wash buffer with BD Horizon™ Brilliant Stain Buffer Plus (10 μl per test) for 30 min at 4°C in the dark. Cells were then washed four times with 1 × BD Perm/Wash buffer by centrifugation (500 × g, 5 min, RT), resuspended in 200 μl of staining buffer to be analyzed on a BD FACSymphony™ A5.2 cell analyzer (BD Biosciences). Samples were stained and analyzed in five batches and application settings were applied for each acquisition to ensure consistency in the instrument performance.

### Data Analysis Workflow

Flow data were analyzed using CATALYST (28), explored using tidyverse (29) and visualized using ggplot and pheatmap (30) in the R statistical environment (31).

Briefly, flow cytometry files (fcs files) were pre-processed [compensated, gated for single and viable cells and quality control checked using FlowAI (32)]. Data was then scaled and exported for analysis using R (v4.0). FlowSOM (33) is an algorithm for clustering cells with Self-Organizing Maps (SOMs), based on the behavior of chosen markers on all cells. We used FlowSOM to cluster cells (max k = 50, using 20 × 20 SOM grid) by surface markers including CD3, CD4, CD8, CD19, CD25, CD127, CD11c, CD14, CD16, CD56, HLA-DR, which were referred to as “type markers”. The type markers are lineage markers that were used to define cell types within PBMCs. Clusters were then manually annotated based on dominant cell frequency. Besides the type markers, we chose eight markers, including IL-2, IL-6, IL-10, IFN-γ, TNF-α, IL-17A, CCL-4, and HLA-DR, to be the “state markers,” because expression levels of these state markers give indications on the functional state of the cell. Uniform Manifold Approximation and Projections (UMAPs) were generated based on type or state marker expression, limited to 3,000 cells per sample. We also performed Differential State (DS) analysis using a modified version of diffcyt (34). For DS, cluster medians for state markers were extracted, background corrected (>0 in more than half the number of samples in the smallest group) and then compared using the limma eBayes test (35) with customized contrast matrices and multiple testing (corrected *p*-value cut-off of 0.05). Marker list overlaps were visualized using euler diagrams (36).

## Results

### Experimental Design and Workflow

**Figure 1:**
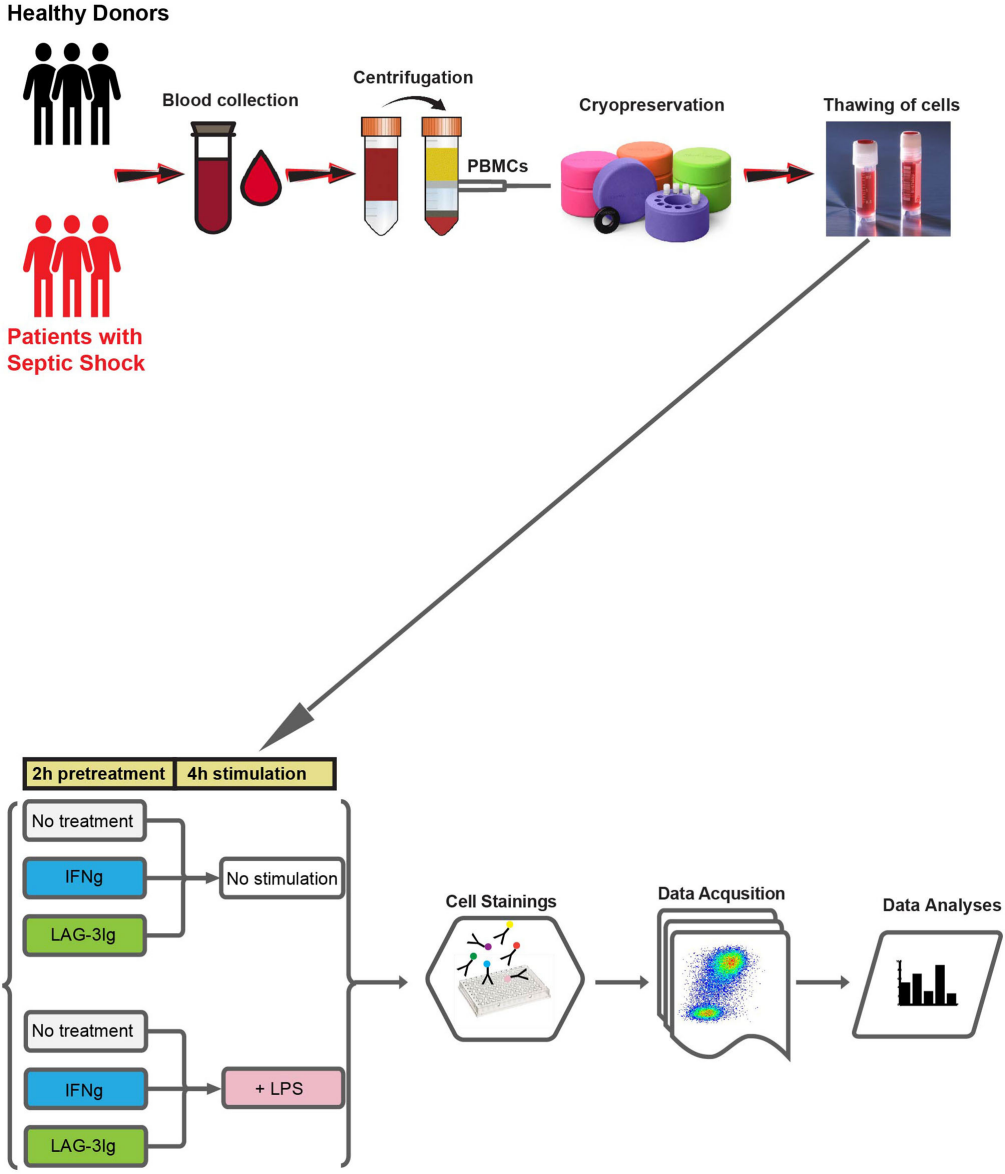
Outline of experimental design and workflow. Whole blood samples are collected from either healthy donors or patients with septic shock. PBMCs are prepared from whole blood using Ficoll gradient centrifugation and cryopreserved. On the day of the experiment, PBMCs are thawed and subjected to six different culture conditions: (1) 2 hours (2 h) “No treatment” followed by 4 hours (4 h) “No stimulation” (No treatment + No stimulation); (2) IFN-γ + No stimulation; (3) LAG-3Ig + No stimulation; (4) LPS stimulation; (5) IFN-γ + LPS stimulation; (6) LAG-3Ig + LPS stimulation. After 6 h of culture, cells are stained with fluorochrome conjugated antibodies, followed by data acquisition using BD FACSymphony™ A5.2 cell analyzer (BD Biosciences). Finally, data is subjected to high-resolution clustering and empirical Bayes moderated tests adapted from transcriptomics.

Figure 1 outlines the experimental design and workflow. PBMCs isolated from the whole blood of either healthy donors or patients with septic shock, underwent six different pre-treatment and/or stimulation conditions before they were stained and subjected to flow cytometry analysis.

### Immune Cell Composition Is Significantly Altered in Sepsis

**Figure 2:**
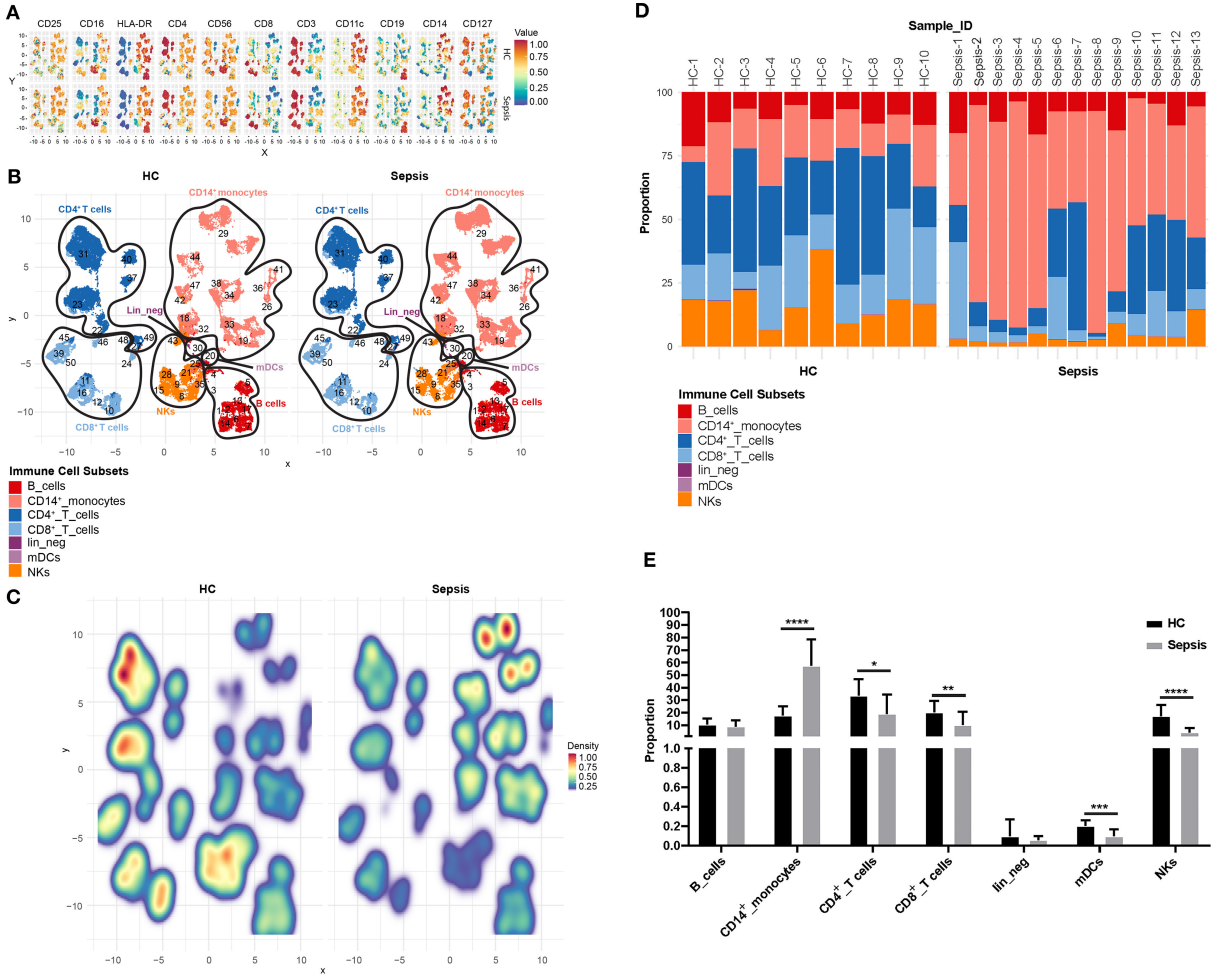
Proportion of immune cell subsets in PBMCs. **(A)** Uniform Manifold Approximation and Projection (UMAP) displays the median expression levels of all the type markers on total untreated PBMCs (no treatment or LPS stimulation) in HC and sepsis. **(B)** 50 meta-clusters, as defined by dominant cell frequency, are shown on UMAP. Based on the presence or absence of specific type markers as shown in **(A)**, the 50 meta-clusters are annotated into seven immune cell subsets. **(C)** Relative proportions of the meta-clusters in PBMCs are compared between HC and sepsis. **(D)** Proportions of the seven immune cell subsets in PBMCs are shown in 10 HC (HC-1 to HC-10) and 13 Sepsis patients (Sepsis-1 to Sepsis-13). Values from each subject were plotted. **(E)** Bar diagram represents mean proportion of each immune cell subset as indicated on the X-axis in HC vs. sepsis. Data represents mean ± SD; *P < 0.05, **P < 0.01, ***P < 0.001, ****P < 0.0001, were determined by Mann-Whitney test.

In order to compare immune cell composition of PBMCs between healthy controls (HC, *n* = 10) and sepsis patients (sepsis, *n* = 13), flow cytometric data from untreated PBMCs (no treatment or LPS stimulation) from both HC and sepsis groups, were concatenated and FlowSOM was used to cluster cells by “type markers” as detailed in the materials and methods. Median expression levels of all the type markers in both HC and sepsis groups were shown on the UMAPs (Figure 2A). Based on dominant cell frequency, 50 meta-clusters were defined (Figure 2B). The meta-clusters were then merged into seven different immune cell subsets: CD19^+^ B cells, CD14^+^ monocytes, CD3^+^CD4^+^ T cells, CD3^+^CD8^+^ T cells, CD11c^+^ mDCs, CD3^−^CD56^+^ NK cells, and lineage negative (lin_neg) cells, based on the presence or absence of specific type markers. Relative proportions of the seven immune cell subsets in PBMCs were compared between HC and sepsis (Figure 2C). Our results demonstrate the stark differences between the immune profile of HC and Sepsis subjects. There was a trend toward increased proportion of CD14^+^ monocytes and decreased proportions of CD4^+^, CD8^+^ T cells, and NK cells in sepsis as compared to HC. Proportions of the seven immune cell subsets in individual sample were shown in Figure 2D. Heterogeneity within the sepsis patients was observed. Among the seven immune cell subsets investigated, proportions of CD14^+^ monocytes, CD4^+^ T cells, CD8^+^ T cells, mDCs, and NKs were significantly altered in sepsis compared to HC (Figure 2E).

### Subgroups in Sepsis: Hyper vs. Hypo

**Figure 3:**
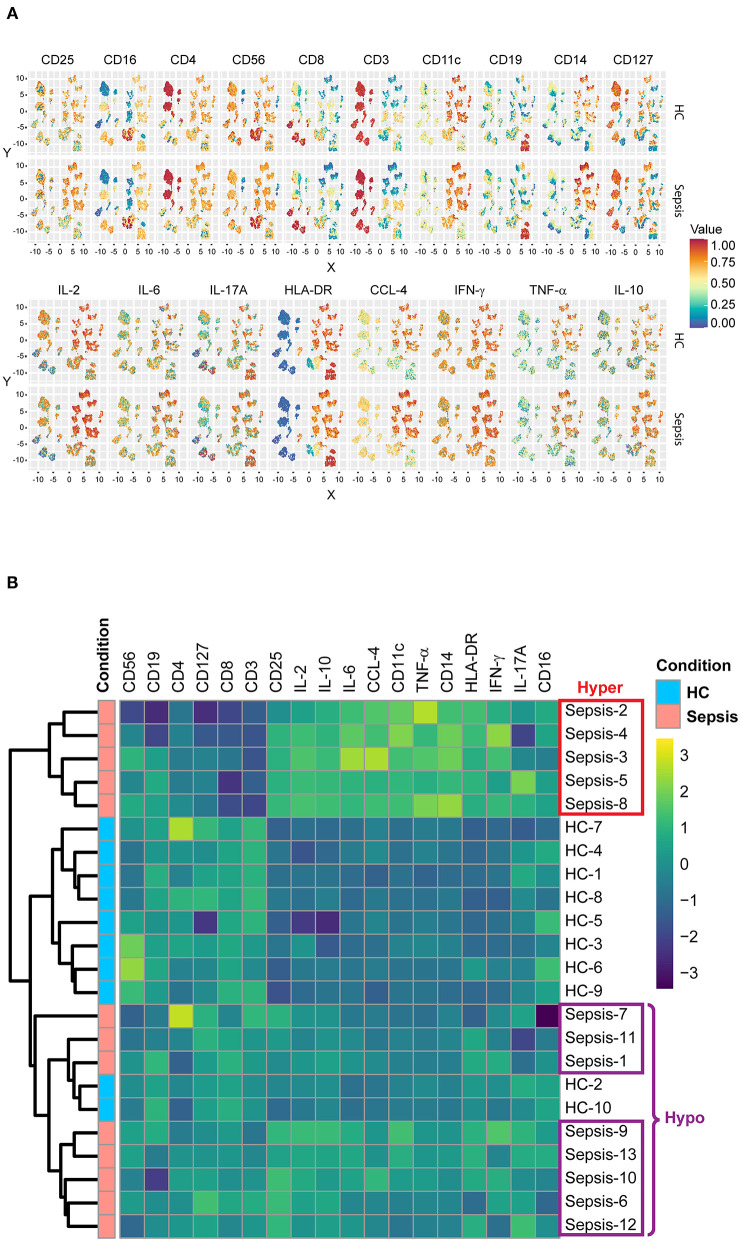
Subgroups in sepsis. **(A)** UMAP displays the median expression levels of all the type and state markers on total PBMCs after 4 h of LPS stimulation without pretreatment in HC and sepsis. **(B)** Heatmap represents the hierarchical clusters based on the median expression levels of all the type and state markers as in **(A)**. Five samples from sepsis group (Sepsis-2, 3, 4, 5, and 8) formed a cluster named as “Hyper” subgroup and the other eight samples (Sepsis-1, 6, 7, 9, 10, 11, 12, and 13) formed a different cluster named as “Hypo” subgroup.

Median expression levels of all the “type” and “state markers” (as detailed in the materials and methods) on total PBMCs after 4 h of LPS stimulation without pretreatment with IFN-γ or LAG-3Ig, were compared across all the meta-clusters between HC and sepsis as displayed on UMAPs (Figure 3A). We observed an overall trend toward decreased HLA-DR expression on CD14^+^ monocytes meta-clusters in sepsis as compared to HC, which is one of the hallmarks for sepsis. Median expression levels of all the markers in the seven immune cell subsets were compared between HC and sepsis (Supplementary Figure 1). To explore if there was any presence of subgroups in the heterogeneity observed in Figure 2D, we performed hierarchical clustering analysis, an algorithm used to group similar objects into clusters with each cluster distinct from each other. Median expression levels of all the type and state markers as in Figure 3A, were used for the hierarchical clustering analysis. Eight out of the 10 HC samples formed a tight cluster whereas sepsis samples were segregated into two subgroups which we named “Hyper” and “Hypo” subgroups (Figure 3B). The Hyper subgroup displayed an overall upregulation of surface markers CD25, CD11c, CD14, HLA-DR, and intracellular cytokines and chemokine as well as down regulated expression of CD3, CD8, CD127, and CD4 compared to HC. In the Hypo subgroup, expression of CD25, CD11c, CD14, HLA-DR, and intracellular cytokines and chemokines was intermediate between HC and the Hyper subgroup whereas expression of CD3, CD8, CD127, and CD4 was similar to that in the HC.

### Commonality and Difference Between the “Hyper” and “Hypo” Subgroups

**Figure 4:**
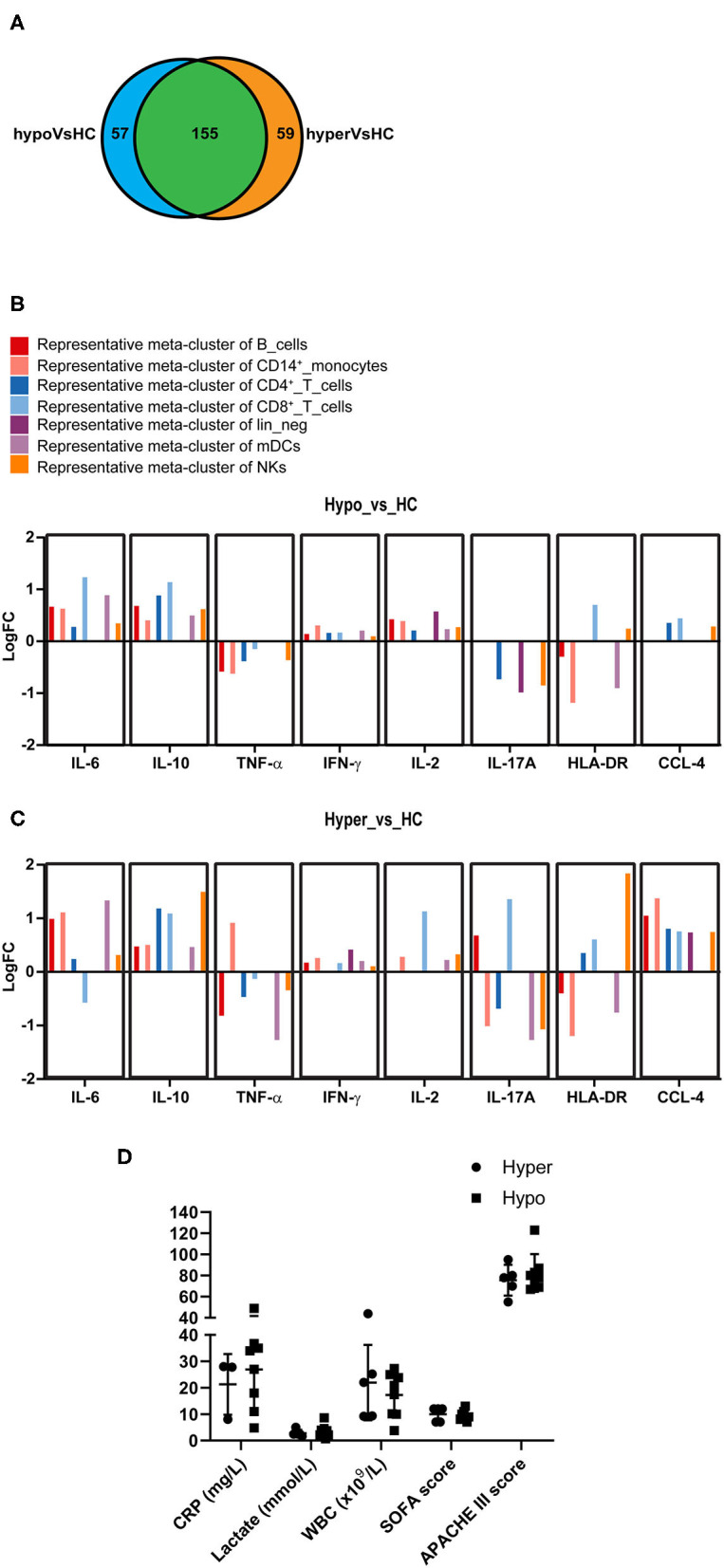
Commonality and difference between the Hyper and the Hypo subgroups. **(A)** Venn diagram represents the number of state markers across 50 meta-clusters of PBMCs that are significantly different between Hyper and HC (in orange) or between Hypo and HC (in blue). The area highlighted in green represents changes that are found in both Hyper and Hypo subgroups compared to HC. **(B)** Bar diagram represents fold changes (logFC) at the expression levels of eight state markers that are significantly altered in Hypo subgroup compared to HC. A representative cluster is shown. **(C)** Bar diagram represents fold changes (logFC) at the expression levels of eight state markers that are significantly altered in Hyper subgroup compared to HC. A representative cluster is shown. **(D)** C-reactive protein (CRP), serum lactate level, whole blood count (WBC), Sequential Organ Failure Assessment (SOFA) score and Acute Physiology and Chronic Health Evaluation (APACHE) III score are compared between the Hyper and the Hypo subgroups. Data represents mean ± SD and *P* < 0.05 is considered significant as determined by Mann-Whitney test.

To further characterize the immune status of the two subgroups of sepsis, we performed differential state (DS) analysis by comparing Hyper and Hypo subgroups to HC in the expression levels of the state markers. Expressions of the state markers on 50 meta-clusters of PBMCs in response to LPS stimulation were analyzed. When compared to HC, both Hyper and Hypo subgroups were shown to have significant changes in the levels of the state markers across the 50 meta-clusters, 155 of which were shared between the two subgroups (Figure 4A). Significant differences between Hyper and Hypo subgroups were also observed. There were 59 unique immune alterations for the Hyper subgroup and 57 unique immune alterations for the Hypo subgroup. Differential expression of the state markers in Sepsis compared to HC were expressed as Log2 fold changes (LogFC). Representative meta-clusters from each immune cell subset, which had significant LogFC in either of the eight state markers, were shown in Figure 4B comparing the Hypo subgroup to HC (Hypo_vs._HC) and Figure 4C comparing the Hyper subgroup to HC (Hyper_vs._HC). An expanded version of Figures 4B,C can be found in Supplementary Figures 2A,B. Major differences between Hyper and Hypo subgroups were observed in IL-6, TNF-α, IL-17A, HLA-DR, and CCL-4 expression levels. IL-6 production in CD8^+^ T cells was decreased in the Hyper but was increased in the Hypo subgroup. Monocyte expression of TNF-α production was upregulated in the Hyper but downregulated in the Hypo subgroup. IL-17A production was upregulated in CD19^+^ B cells and CD8^+^ T cells from the Hyper but not in the Hypo subgroup. We also observed a more than 2 × fold increase in HLA-DR expression on NKs from the Hyper subgroup, which was absent in the Hypo subgroup. CCL-4 upregulation was observed across most of the immune cell subsets in Hyper subgroup. In the Hypo subgroup, however, CCL-4 expression was only increased slightly in CD4^+^, CD8^+^ T cells, and NKs. On the other hand, IL-10 and IFN-γ expression levels did not vary too much between these two subgroups. In light of the differences in the immune status between the Hyper and the Hypo subgroups, we sought to determine if the clinical characteristics of these two subgroups also differ. C-reactive protein (CRP), serum lactate, whole blood count (WBC), SOFA score, and Acute Physiology and Chronic Health Evaluation (APACHE) III score were compared between the Hyper and the Hypo subgroups (Figure 4D). We did not observe significant differences in these clinical characteristics between the two subgroups. Detailed information on the age and gender of these patients in addition to the above-mentioned clinical data can be found in Supplementary Table 2.

### Differential Responses of Hypo and Hyper Subgroup of Sepsis to IFN-γ or LAG-3Ig

**Figure 5:**
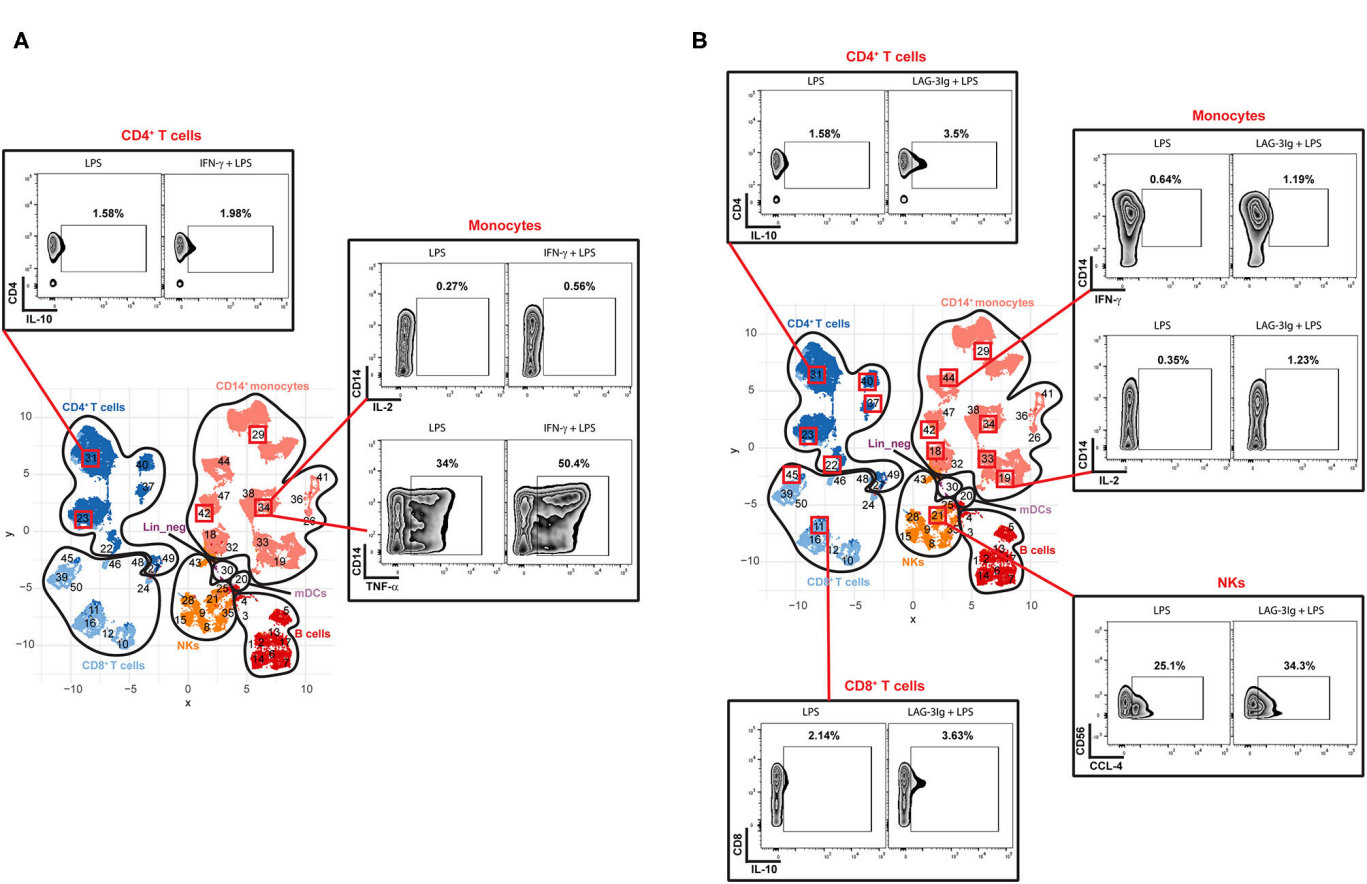
Responses of the Hypo subgroup to IFN-γ or LAG-3Ig pretreatments. UMAP represents the 50 meta-clusters and their corresponding immune cell subsets in sepsis as shown in Figure 2B. Meta-clusters that are showing significant responses to either IFN-γ or LAG-3Ig are squared out in red. Representative flow plots of the representative meta-clusters demonstrate responses of the Hypo subgroup to IFN-γ pretreatment **(A)**, the Hypo subgroup to LAG-3Ig pretreatment **(B)**.

To determine if Hyper and Hypo subgroups would respond differently to IFN-γ or LAG-3Ig, we performed DS analysis within each subgroup by comparing median expression of the state markers under “No treatment + LPS” (LPS) to that under either “IFN-γ pretreatment + LPS” (IFN-γ) or “LAG-3Ig pretreatment + LPS” (LAG-3Ig) conditions. As shown in Figures 5A,B, immune cell subsets from the Hypo subgroup of sepsis showed significant responses to either IFN-γ or LAG-3Ig. Within each cell subset, the meta-clusters which demonstrated significant responses to either treatment were squared out in red. In response to IFN-γ + LPS, three meta-clusters (#29, 34, and 42) within CD14^+^ monocytes from the Hypo subgroup had higher TNF-α expression and meta-cluster #34 also had higher IL-2 expression as compared to no treatment LPS group (Figure 5A). Two meta-clusters (#23 and 31) within CD4^+^ T cells from the Hypo subgroup also responded to IFN-γ by producing more IL-10 as compared to no treatment LPS group. Figure 5B showed responses of the Hypo group to LAG-3Ig treatment as compared to no treatment LPS group. All four meta-clusters of CD4^+^ T cells (#22, 23, 31, 37, and 40), two meta-clusters within CD8^+^ T cells (#11 and 45) responded to LAG-3Ig by producing more IL-10. Seven meta-clusters within CD14+ monocytes (#18, 19, 29, 33, 34, 42, and 44) produced more IFN-γ and IL-2 in response to LAG-3Ig. An increased CCL-4 expression was observed in meta-cluster #21 within NK cells in response to LAG-3Ig. On the other hand, responses of the Hyper subgroup to either IFN-γ or LAG-3Ig were less prominent compared to the Hypo subgroup. The Hyper subgroup demonstrated no significant responses to IFN-γ treatment (data not shown) and minimal responses to LAG-3Ig as shown in Supplementary Figure 3.

## Discussion

The immune response during sepsis is variable and often changes as the disease progresses (4). Many factors determine the immune response such as the nature and virulence of the pathogens, site of infection, patient's genetics, physical condition and co-morbidities. It is therefore crucial to assess individual patient's immune status to personalize any immune-modulatory therapy. However, the complex nature of immune response during sepsis requires a comprehensive analysis to capture changes to the important immune cells and their functions, which may allow accurate identification of patients that would benefit from immune modulating treatment. Current markers of sepsis including WBC, CRP, procalcitonin (PCT) or HLA-DR are insufficient for this purpose. CRP and PCT both indicate the acute phase of inflammatory responses but are not helpful in detecting immunosuppression in septic patients (37–39). Decreased expression of HLA-DR on monocytes is so far the best biomarker for detecting immunosuppression in sepsis (11, 40), but is limited to one cell type and does not provide information on the status of other immune cells. We have also shown in the current study that clinical characteristics such as WBC, CRP, serum lactate, SOFA and APACHE III score, monocytic HLA-DR expression and previous site of infections do not reveal the difference in patient's immune status as demonstrated between the Hyper and the Hypo subgroups (Figure 4D, Supplementary Table 2, Supplementary Figure 4).

In this study, we investigated the potential of HDCyto in revealing and assessing the heterogeneity of immune responses in patients with septic shock. The uniqueness of our HDCyto panel is the incorporation of markers for both enumeration of major immune cell subsets within PBMCs and assessment of functional status of the individual subset. This provided a broad-spectrum clarity on the overall immune status of PBMCs. Importantly, HDCyto analysis tools that were used in this study, including FlowSOM, diffcyt, and CATALYST analysis, provide the means to dissect heterogeneity, and hence identify changes that may be missed if analyzed using 2D approaches, due to the variations within each immune subset. Using this approach, we discovered that immune responses in PBMCs from septic shock patients were significantly altered compared to those from healthy controls, which were observed across all immune cell subsets analyzed. Furthermore, our results revealed, for the first time, two subgroups with distinct immune profiles were present within the sepsis group. The Hyper subgroup was found to have an overall higher production of all state markers in response to *in vitro* LPS stimulation, than the Hypo subgroup. In particular, we observed an increase of HLA-DR expression on NK cells in the Hyper subgroup as compared to the Hypo subgroup. HLA-DR^+^ NK cells have been associated with a less mature phenotype with antigen-presentation ability (41, 42). This might explain the overall hyper-responsiveness as seen in the Hyper subgroup where NKs, in addition to professional antigen-presenting cells such as DCs, leads to further activation of T-cells. Another interesting finding about the Hyper subgroup is the increased IL-17A production by CD19^+^ B cells and CD8^+^ T cells when compared to the Hypo subgroup. Traditionally, IL-17A was thought to be produced mainly by activated Th-17 cells. However, production of IL-17A by other immune cell types such as CD8^+^ T cells and CD19^+^ B cells has been reported previously and has been associated with inflammatory disease (43, 44). IL-17A production by CD8^+^ T cells has also been associated with persistent immune activation in patient with HIV (44). This again explains the hyper-responsiveness as seen in the Hyper subgroup.

We then proceed to assess whether the identification of these two subgroups could help predict responses to immune-modulatory therapy. We evaluated the *ex vivo* effects of immunostimulant agents IFN-γ and LAG-3Ig on PBMCs. Our results showed that the Hyper subgroup did not respond as much as the Hypo subgroup to either IFN-γ or LAG-3Ig in terms of changes in the expression of state markers on different immune cell subsets. Given that the Hyper subgroup already had an elevated basal level of all the state markers compared to the Hypo subgroup, this result is not surprising and suggests that immunostimulant treatment is likely to be ineffective or potentially harmful in the “Hyper” subgroup. We can only speculate whether these patients potentially respond to immune suppressive treatment such as steroids. On the other hand, the PBMCs from the Hypo subgroup, exposed to IFN-γ prior to LPS stimulation, showed increased IL-10 and TNF-α production in CD4^+^ T cells and monocytes. Similarly, exposure to LAG-3Ig also enhanced the production of IL-10 in CD4^+^ and CD8^+^ T cells and CCL-4 production in NKs.

The effects of IFN-γ were seen mainly in monocytes, but not in other cells (Figure 5A), which is likely due to the limited 4 h stimulation with a Toll-like Receptor 4 agonist LPS. Further studies may be required to investigate the effect of IFN-γ over longer periods of stimulation with different stimulus. In comparison to IFN-γ, LAG-3Ig seemed to have pleiotropic effects on different immune cells subsets including monocytes, NKs, CD4^+^ and CD8^+^ T cells (Figure 5B). Whether or not sepsis patients with “Hypo-like” immune status will benefit from immunostimulant therapy such as IFN-γ or LAG-3Ig, remains to be investigated further.

This study using HDCyto provides a deeper understanding of the altered immune phenotype in severe sepsis, providing a window to future therapies. Our small study demonstrated the feasibility and advantage of using HDCyto in assessing immune signature of sepsis patients. It is important to note that many of our findings are in line with previous studies (8, 11, 22, 29, 45), with our panel allowing for assessments in numerous immune cell subsets at the same time. Being able to group patients based on the overall immune status is an important finding. We also demonstrated that the identified groups differed in their response to pretreatment with immune modulatory agents. Further investigations on various *ex vivo* stimulations followed by HDCyto analyses in monitoring patient's immune status and their response to immunomodulatory treatments such as IFN-γ, immune checkpoint inhibitors, tocilizumab and steroids, may allow clinician to tailor sepsis treatments. The protocol for the assessment of intracellular cytokines in this study requires hours of stimulation before measurements. Future studies may aim to find surface markers that could be used as surrogates for immune cell functions, such as CD69, CD80, CD86, PD-1, PD-L1, or LAG-3 among others. This would be critical for timely clinical application of HDCyto in triaging sepsis patients promptly before receiving immunotherapies.

## Data Availability Statement

The raw data supporting the conclusions of this article will be made available by the authors, without undue reservation.

## Ethics Statement

The studies involving human participants were reviewed and approved by Nepean Blue Mountains Local Health District Human Research Ethics Committee. The patients/participants provided their written informed consent to participate in this study.

## Author Contributions

BG was responsible for data analysis and generating figures. BT, DB, and AM were responsible for study concept and manuscript revision. SD was involved in flow panel design and data analysis. CB, FT, and BS-N provided advice on the experiment design. SS and NF provided technical advice on flow cytometry. CW, KM, KS, and ST were responsible for recruiting subjects, sample processing, and ethics application. YW was responsible for flow panel design and optimization, data acquisition, data analysis, and manuscript writing. MN was responsible for study concept, design, ethics application, subject recruitment, data analysis, and interpretation. Final approval for submission was given by all authors.

## Conflict of Interest

CB and BT were employed by Immutep S.A.S. The remaining authors declare that the research was conducted in the absence of any commercial or financial relationships that could be construed as a potential conflict of interest.

## Supplementary Material

Supplementary Figure 1 Heatmap represents the median expression levels of all the type and state markers in the seven immune cell subsets (as shown in Figure 2B) on total PBMCs after 4 h of LPS stimulation without pretreatment in HC and Sepsis.

Supplementary Figure 2 **(A,B)** Are the expanded version of Figures 4B,C, respectively. The expanded version includes all meta-clusters from each immune subset, which show significant changes in the expression levels of the state marker when comparing the Hypo to HC as in **(A)** or comparing the Hyper to HC as in **(B)**. Meta-cluster with a “^*^” is the representative meta-cluster of the immune subset as indicated by the color code. The representative meta-cluster was chosen based on its logFC, which is either the median of all included meta-clusters (if more than two meta-clusters) or the average of two meta-clusters.

Supplementary Figure 3 Responses of the Hyper subgroup to LAG-3Ig pretreatment. UMAP represents the 50 meta-clusters and their corresponding immune cell subsets in sepsis as shown in Figure 2B. Meta-clusters that are showing significant responses to either LAG-3Ig are squared out in red. Representative flow plots of the representative meta-clusters demonstrate responses of the Hyper subgroup to LAG-3Ig pretreatment.

Supplementary Figure 4 Additional clinical characteristics are compared between the Hyper and the Hypo subgroups. **(A)** Whole blood count (WBC) (×10^9^/L) as well as the counts for neutrophils, monocytes and lymphocytes are compared between the Hyper and the Hypo subgroups. *P*-values were determined by Mann-Whitney test. **(B)** Median Fluorescence Intensity (MFI) of HLA-DR on CD14^+^ monocytes are compared among the three groups: HC, the Hypo and Hyper subgroups. *P*-values were determined by Ordinary One-Way ANOVA—Dunnett's multiple comparison test. **(C)** Table shows details on the site of infections for patients from the Hyper and the Hypo subgroups. Data represents mean ± SD, ^****^*p* < 0.0001.

Supplementary Table 1 Names of antigens and cell types detected by the HDCyto flow panel are listed. Fluorochrome and clone number of each antibody are also listed.

Supplementary Table 2 Clinical characteristics of patients from the Hyper and the Hypo subgroups. Age, Male sex%, C-reactive protein (CRP) (mg/L), Serum lactate (mmol/L), SOFA score, whole blood count (WBC) (×10^9^/L), and Acute Physiology and Chronic Health Evaluation (APACHE) III score are compared between the Hyper and the Hypo subgroups. *P*-values were determined by Mann-Whitney test.
