# Supplementary figures and images for: Immunophenotyping of Peripheral Blood Mononuclear Cells in Septic Shock Patients With High-Dimensional Flow Cytometry Analysis Reveals Two Subgroups With Differential Responses to Immunostimulant Drugs
Source: Front Immunol. 2021 Mar 22;12:634127. doi: 10.3389/fimmu.2021.634127 (PMC8019919; doi:10.3389/fimmu.2021.634127)

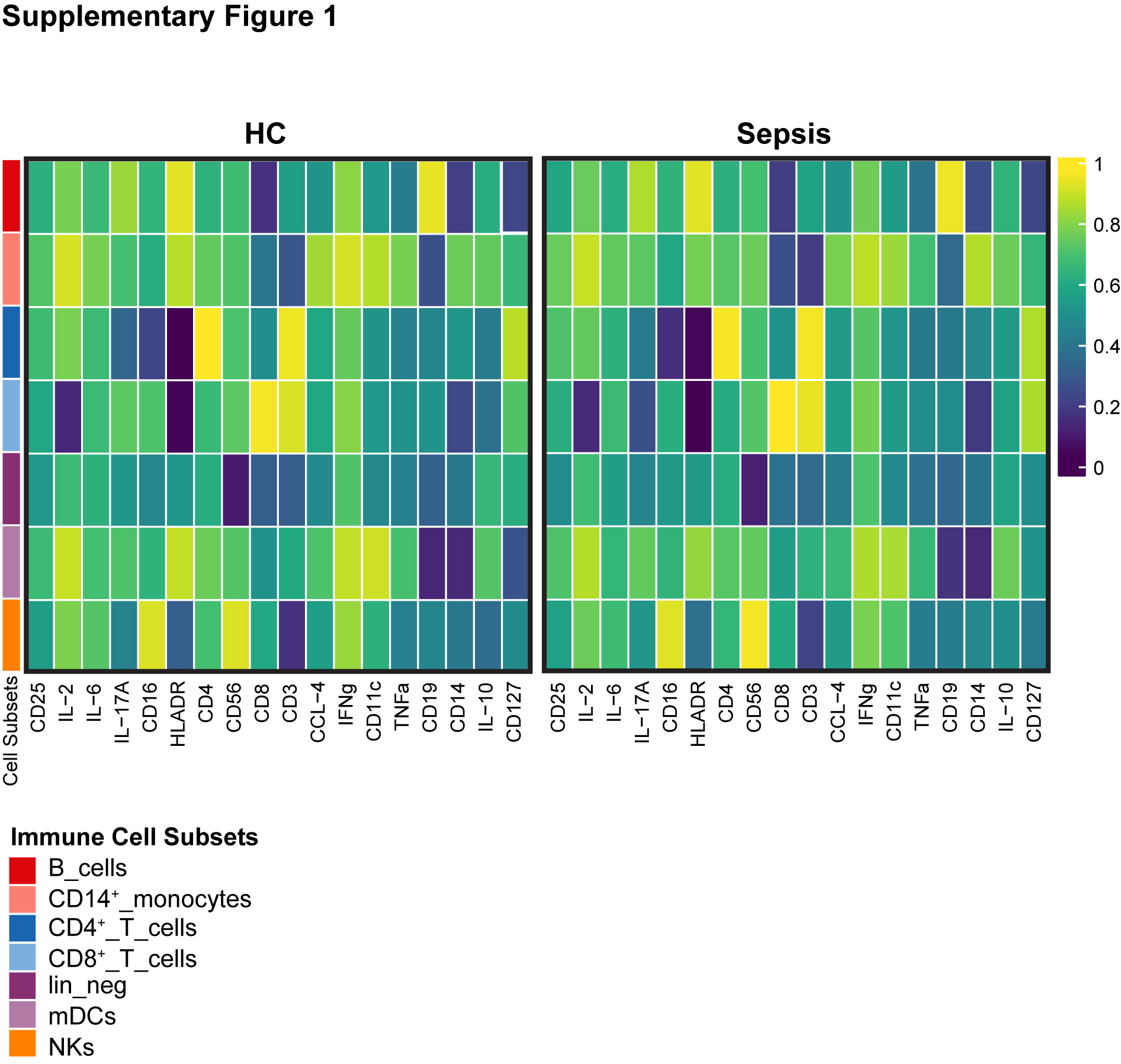

Supplement: Supplementary Figure 1 — Heatmap represents the median expression levels of all the type and state markers in the seven immune cell subsets (as shown in Figure 2B) on total PBMCs after 4 h of LPS stimulation without pretreatment in HC and Sepsis. [file Image_1.TIF]

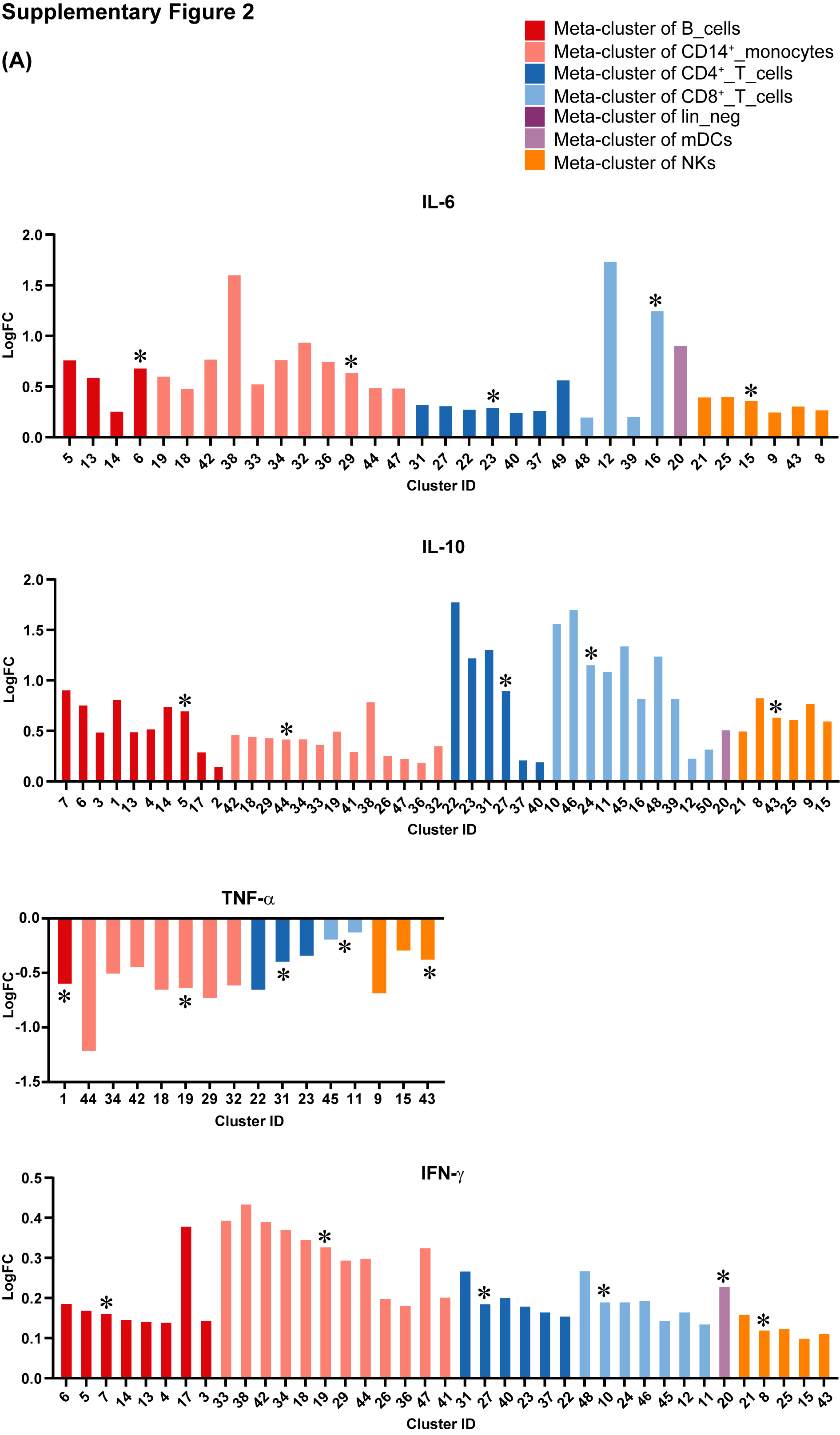

Supplement: Supplementary Figure 2 — (A,B) Are the expanded version of Figures 4B,C, respectively. The expanded version includes all meta-clusters from each immune subset, which show significant changes in the expression levels of the state marker when comparing the Hypo to HC as in (A) or comparing the Hyper to HC as in (B). Meta-cluster with a “*” is the representative meta-cluster of the immune subset as indicated by the color code. The representative meta-cluster was chosen based on its logFC, which is either the median of all included meta-clusters (if more than two meta-clusters) or the average of two meta-clusters. [file Image_2.TIF]

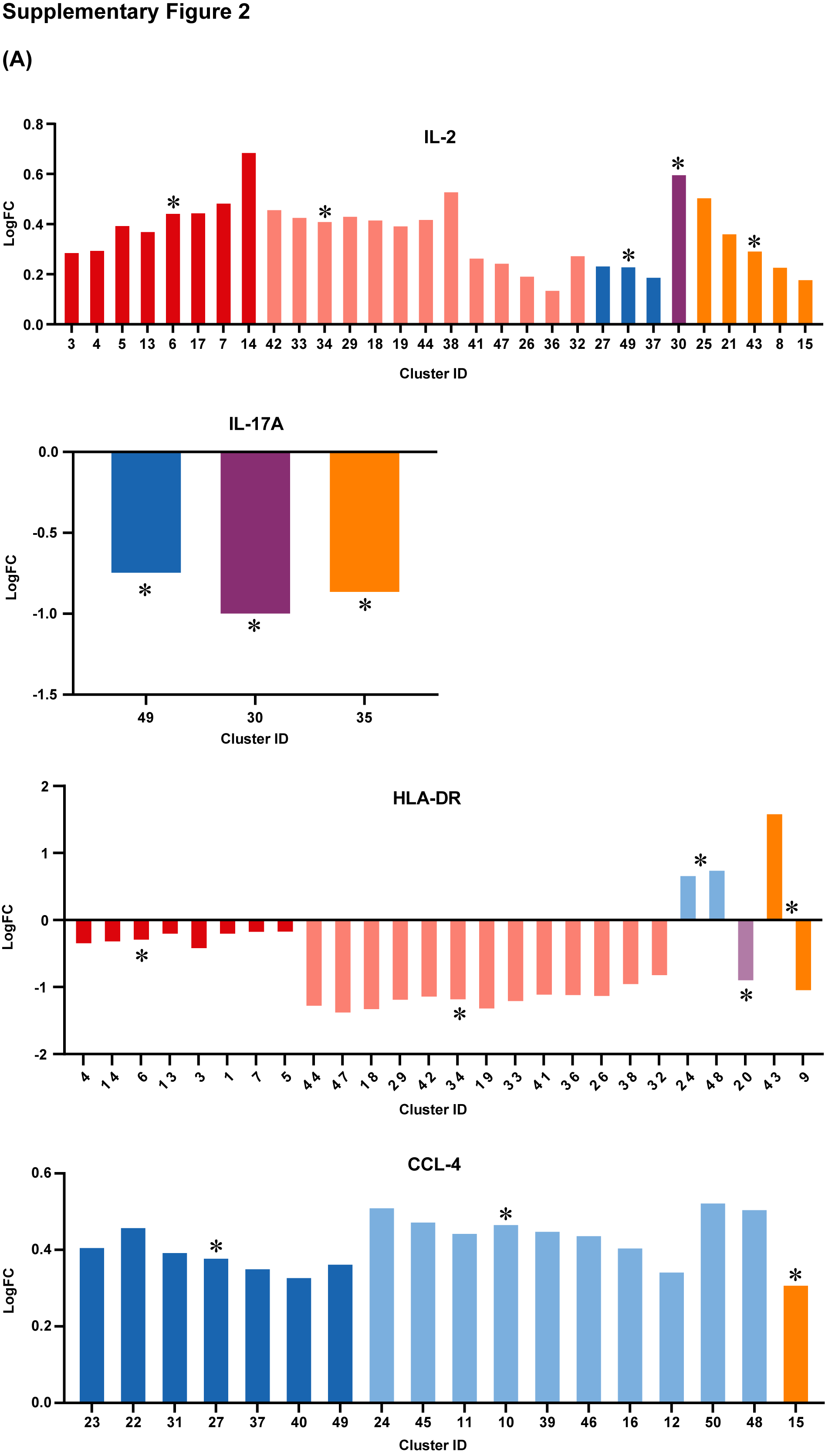

Supplement: Supplementary file 3 [file Image_3.TIF]

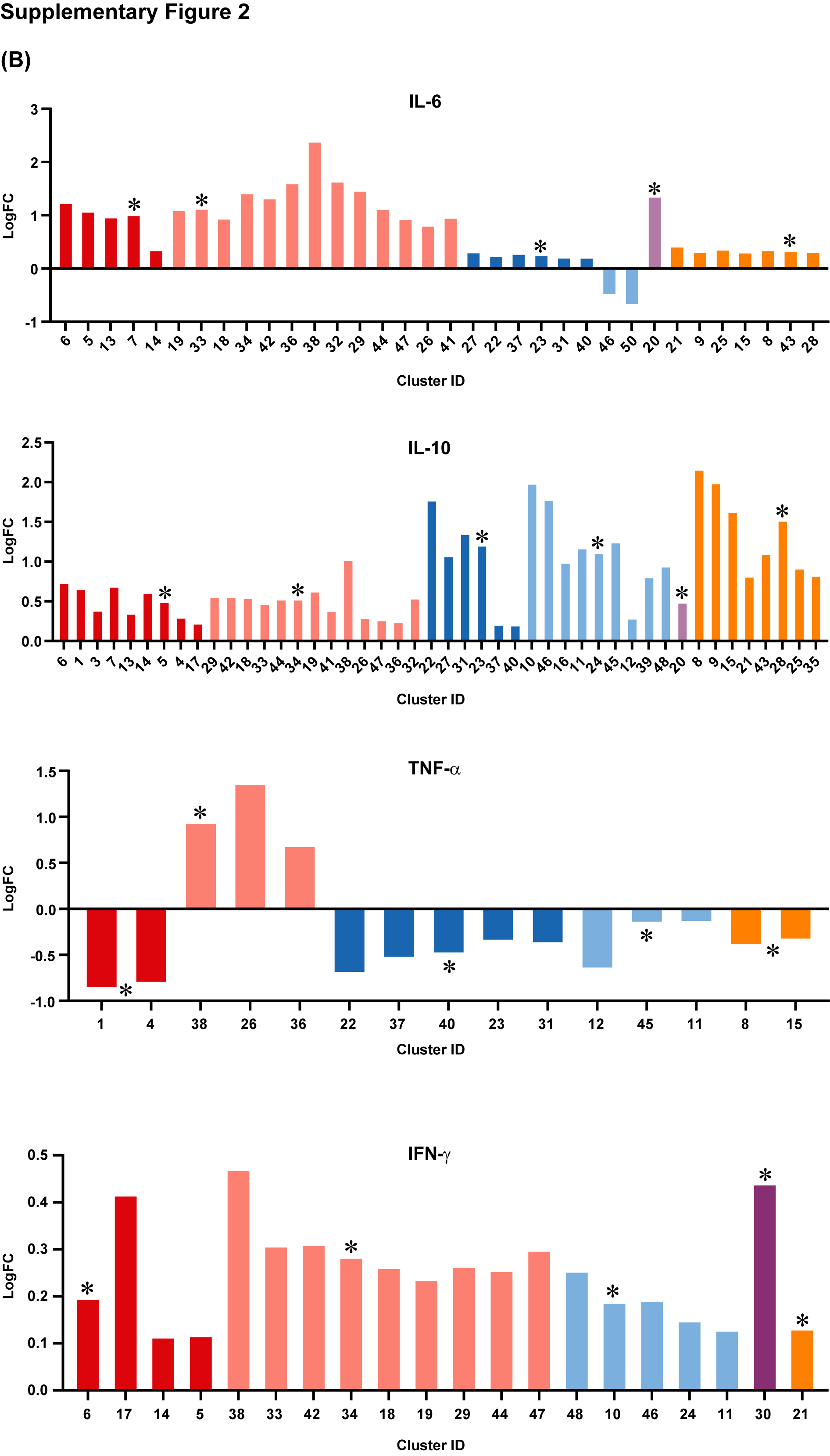

Supplement: Supplementary file 4 [file Image_4.TIF]

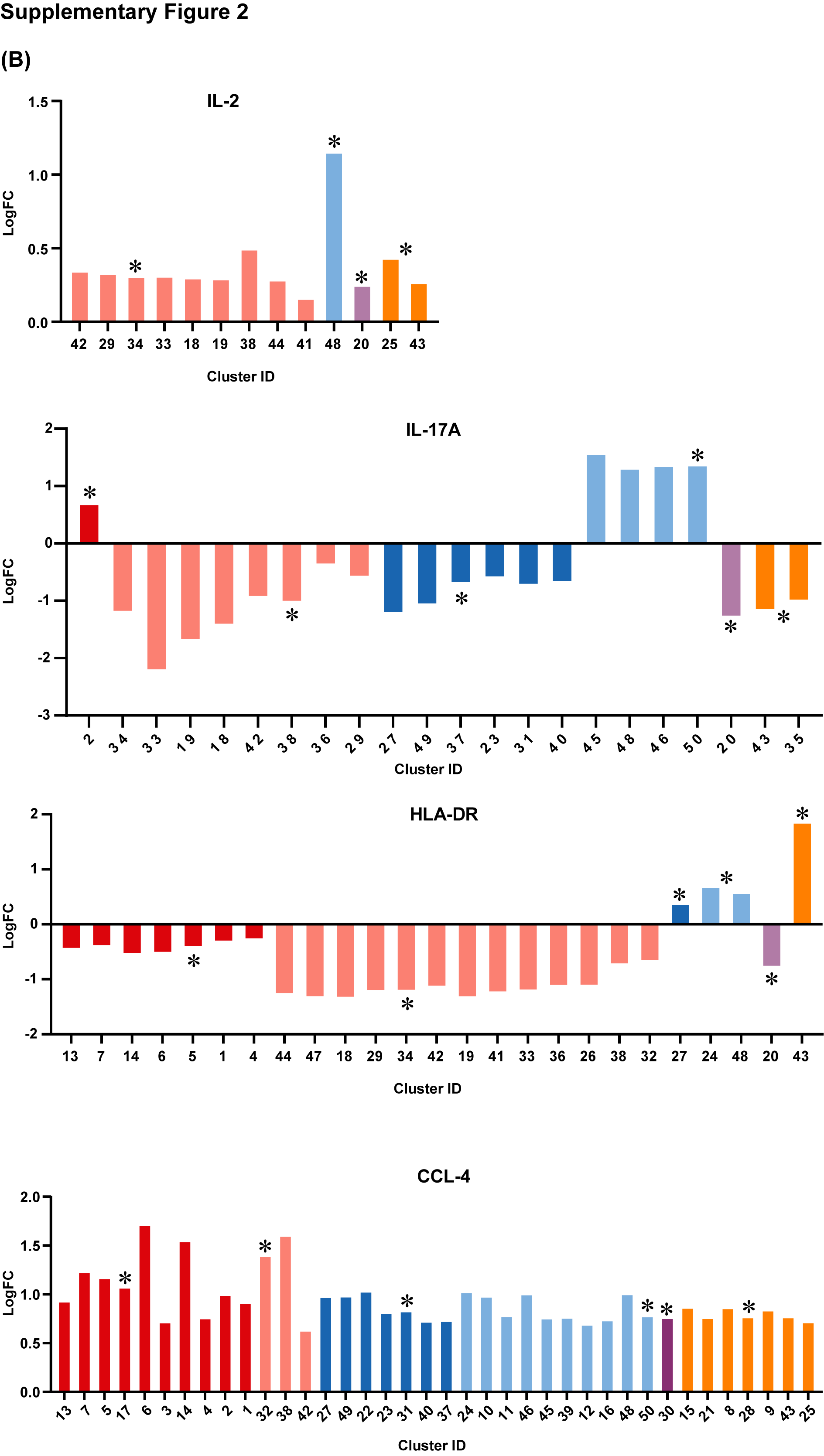

Supplement: Supplementary file 5 [file Image_5.TIF]

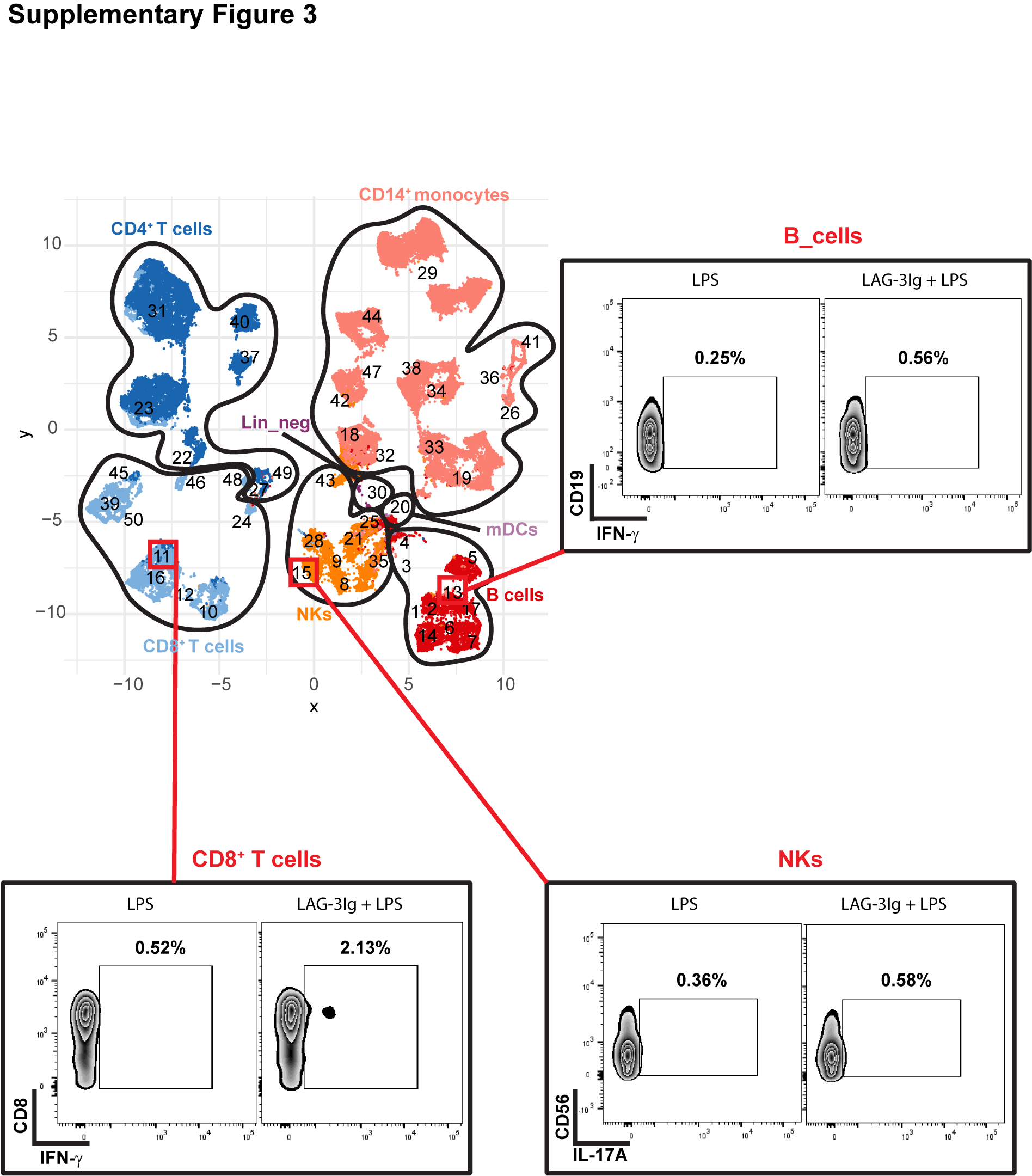

Supplement: Supplementary Figure 3 — Responses of the Hyper subgroup to LAG-3Ig pretreatment. UMAP represents the 50 meta-clusters and their corresponding immune cell subsets in sepsis as shown in Figure 2B. Meta-clusters that are showing significant responses to either LAG-3Ig are squared out in red. Representative flow plots of the representative meta-clusters demonstrate responses of the Hyper subgroup to LAG-3Ig pretreatment. [file Image_6.TIF]

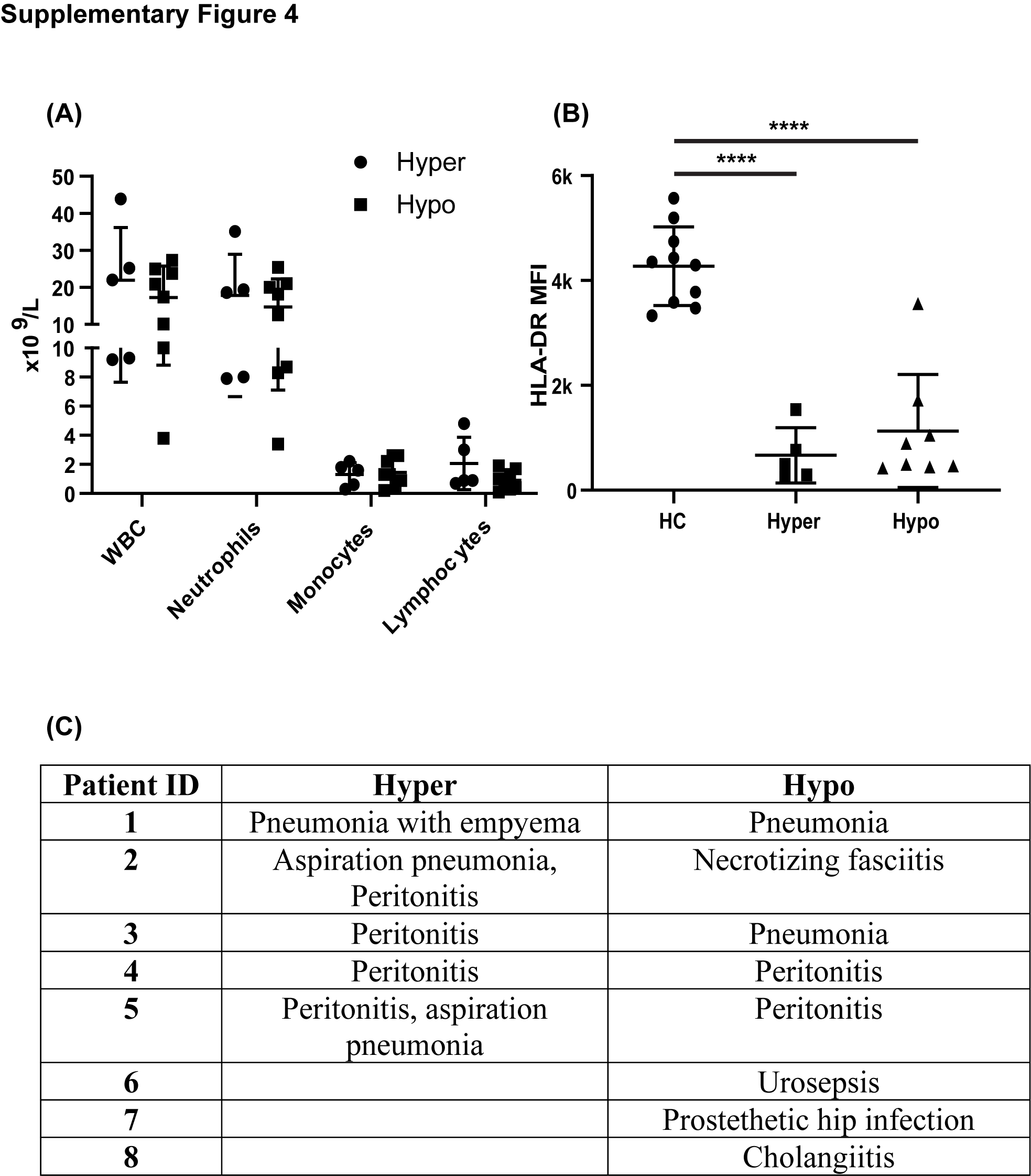

Supplement: Supplementary Figure 4 — Additional clinical characteristics are compared between the Hyper and the Hypo subgroups. (A) Whole blood count (WBC) (×109/L) as well as the counts for neutrophils, monocytes and lymphocytes are compared between the Hyper and the Hypo subgroups. P-values were determined by Mann-Whitney test. (B) Median Fluorescence Intensity (MFI) of HLA-DR on CD14+ monocytes are compared among the three groups: HC, the Hypo and Hyper subgroups. P-values were determined by Ordinary One-Way ANOVA—Dunnett's multiple comparison test. (C) Table shows details on the site of infections for patients from the Hyper and the Hypo subgroups. Data represents mean ± SD, ****p < 0.0001. [file Image_7.TIF]
