# Supplementary material for: Immunophenotyping of Peripheral Blood Mononuclear Cells in Septic Shock Patients With High-Dimensional Flow Cytometry Analysis Reveals Two Subgroups With Differential Responses to Immunostimulant Drugs
Source: Front Immunol. 2021 Mar 22;12:634127. doi: 10.3389/fimmu.2021.634127 (PMC8019919; doi:10.3389/fimmu.2021.634127)
Supplement: Supplementary Table 1 — Names of antigens and cell types detected by the HDCyto flow panel are listed. Fluorochrome and clone number of each antibody are also listed. [file Table_1.DOCX]

**Supplementary Table 1:**

**19-color flow panel for immune profiling of human peripheral blood mononuclear cells**

| Antigen Specificity | Fluorochrome | Clone | Cell Types/functional types |
| --- | --- | --- | --- |
| CD3 | BUV805 | UCHT1 | T cells |
| CD4 | BUV496 | SK3 | T cells |
| CD8 | BUV737 | SK1 | T cells |
| CD25 | BB515 | M-A251 | T cells |
| CD127 | PE-Cy7 | HIL-7R-M21 | T cells |
| CD19 | BV711 | SJ25C1 | B cells |
| CD56 | BUV563 | NCAM16.2 | NKs |
| CD11c | BV605 | B-Ly6 | mDCs |
| CD14 | BV786 | M5E2 | monocytes |
| CD16 | APC-H7 | 3G8 | monocytes |
| HLA-DR | BUV395 | G46-6 | Antigen-presenting cells |
| CCL4 (MIP-1b) | BV421 | D21-1351 | Chemokine ligands 4 |
| IFN-γ | BV480 | B27 | Th1 cytokine |
| TNF-α | BV650 | Mab11 | Th1 cytokine |
| IL-6 | APC | MQ2-12A5 | Tfh cytokine |
| IL-10 | PE | JES3-907 | Th2 cytokine |
| IL-2 | BB700 | MQ1-17H12 | Th1 cytokine |
| IL-17A | APC-R700 | N49-653 | Th17 cytokine |
| Viability | Fixable Viability Stain 440UV (FVS 440UV) |  | Live/dead |
