# Supplementary material for: Immunophenotyping of Peripheral Blood Mononuclear Cells in Septic Shock Patients With High-Dimensional Flow Cytometry Analysis Reveals Two Subgroups With Differential Responses to Immunostimulant Drugs
Source: Front Immunol. 2021 Mar 22;12:634127. doi: 10.3389/fimmu.2021.634127 (PMC8019919; doi:10.3389/fimmu.2021.634127)
Supplement: Supplementary Table 2 — Clinical characteristics of patients from the Hyper and the Hypo subgroups. Age, Male sex%, C-reactive protein (CRP) (mg/L), Serum lactate (mmol/L), SOFA score, whole blood count (WBC) (×109/L), and Acute Physiology and Chronic Health Evaluation (APACHE) III score are compared between the Hyper and the Hypo subgroups. P-values were determined by Mann-Whitney test. [file Table_2.docx]

**Supplementary Table 2**.

**Patient characteristics and clinical features**

|  | **All**  **(n = 13)** | **Hyper**  **(n = 5)** | **Hypo**  **(n = 8)** | **P Value**  **(Hyper vs Hypo)** |
| --- | --- | --- | --- | --- |
| **Age, median (range)** | 69 (46-77) | 70 (46-70) | 65 (49-77) | 0.7467 |
| **Male sex, number (%)** | 8 (62%) | 4 (80%) | 4 (50%) | 0.5649 |
| **C-reactive protein, median (mg/L)** | 27.8 | 27.8 | 30.5 | 0.6303 |
| **Serum lactate, median (mmol/L)** | 2.4 | 2.4 | 3.6 | 0.7242 |
| **SOFA score, median** | 9 | 11 | 9 | 0.8904 |
| **WBC, median, (x10^9^/L)** | 20.9 | 22 | 19.15 | 0.8329 |
| **APACHE III Score** | 78 | 78 | 79 | 0.6977 |
